# International Differences in the Links between Obesity and Physiological Dysregulation: The United States, England, and Taiwan

**DOI:** 10.1155/2013/618056

**Published:** 2013-05-28

**Authors:** Sarinnapha Vasunilashorn, Jung Ki Kim, Eileen M. Crimmins

**Affiliations:** ^1^Office of Population Research, Princeton University, Princeton, NJ 08544, USA; ^2^Davis School of Gerontology, University of Southern California, Los Angeles, CA 90089, USA

## Abstract

Excess weight has generally been associated with adverse health outcomes; however, the link between overweight and health outcomes may vary with socioeconomic, cultural, and epidemiological conditions. We examine associations of weight with indicators of biological risk in three nationally representative populations: the US National Health and Nutrition Examination Survey, the English Longitudinal Study of Ageing, and the Social Environment and Biomarkers of Aging Study in Taiwan. Indicators of biological risk were compared for obese (defined using body mass index (BMI) and waist circumference) and normal weight individuals aged 54+. Generally, obesity in England was associated with elevated risk for more markers examined; obese Americans also had elevated risks except that they did not have elevated blood pressure (BP). Including waist circumference in our consideration of BMI indicated different links between obesity and waist size across countries; we found higher physiological dysregulation among those with high waist but normal BMI compared to those with normal waist and normal BMI. Americans had the highest levels of biological risk in all weight/waist groups. Cross-country variation in biological risk associated with obesity may reflect differences in health behaviors, lifestyle, medication use, and culture.

## 1. Introduction

Rising levels of obesity are becoming a worldwide phenomenon and are increasingly identified as a health problem across the globe [1–4]. Higher weight has been associated with adverse health indicators and outcomes, including cardiovascular disease [5–12], stroke [5, 13], cognitive and functional decline [14–18], metabolic syndrome [19, 20], inflammation [21, 22], and mortality [20, 23–25]. Obesity among aging populations is relatively recent and aging among people who have been obese for much of their lives is also a new phenomenon [26]. From 1980 to 2004, the prevalence of obesity in the US has continued to rise from about 17% to 25% for men aged 50–59. While obesity in England has also increased during this period, from approximately 9% in 1980 to 15% in 2004 for men aged 55–64, the level of obesity remains much lower in England [26]. Additionally, the difference in obesity between the US and England is more pronounced for women. The level of obesity in US women was about 24% in 1980 and rose to 37% in 2004 (age 50–59); in England, levels for women were 9% in 1980 and 14% in 2004 (age 55–64). The aim of this paper is to investigate differences in how obesity relates to indicators of physiological dysregulation in men and women of diverse populations. This comparison will lead to an improved understanding of how obesity might be differentially related to health and mortality across cultures and lifestyles.

Obesity was relatively rare in most populations until the second part of the last century, but it has now become common in many countries [27]. The US population is recognized as among the most obese in the world, although many other countries are now approaching the US level and most countries are experiencing increasing obesity. This worldwide obesity epidemic began with the epidemiological revolution and the virtual elimination of infectious disease; the decline in manual labor needed to provide sustenance with the industrial revolution; and the increasing availability and decreasing cost of food [27–29].

Obesity may result from behavioral differences across cultures or among individuals within cultures. Obesity reflects some combination of calorie intake, diet content, and amount of physical activity. In some cultures, lack of physical activity can be a more important determinant of obesity; in other cultures, overeating or food composition may be the more important determinant of obesity. It is also true that within countries, individuals could differ in the causes of obesity. For instance, changes in activity might be more characteristic of women or men resulting in different reasons for obesity by gender.

These differences may affect how obesity is related to other risk factors for poor health, and it may determine the overall health risk associated with obesity. If physical activity is maintained, the overall effect of obesity may be less than if the activity is not. One indication of the cause of obesity may be the relationship between waist size and weight [30, 31]. In societies where obese people are more physically active, waist size of the obese may be smaller than where physical activity levels are lower. Waist circumference has also been linked to late life mortality, where high waist circumference has been associated with increased mortality among men and women in The Netherlands, while high BMI was not associated with mortality [32]. This paper builds upon current obesity research by using both BMI and waist circumference to quantify obesity in order to determine how a combined indicator of weight and adiposity is related to physiological dysregulation in populations with different cultures, diets, behaviors, and epidemiological histories.

Obesity has been related to many indicators of physiological dysregulation including cardiovascular risk factors such as hypertension [33] and metabolic dysregulation in lipid levels or insulin resistance [34]. Obesity is also related to higher levels of systemic inflammation [35]. Most studies that investigate the differences in biological risk associated with excess weight have examined Western populations [33, 36]. Comparative studies on the health risks associated with obesity that examined the US and England reported that obese Americans had an increased risk of diabetes and a higher waist circumference [37, 38]. These studies suggested that differences in physical activity, diet, and social environments may explain these national differences. While these differences have been observed between the US and England, these two Western countries have roughly similar life expectancy, levels of living, history, and culture even while the US has poorer health by a number of indicators of disease prevalence and biological risk [36, 39].

Comparative studies of the links between obesity and health outcomes and risk factors for obesity comparing Western and non-Western countries indicate important differences in the causes and consequences of obesity. A comparison of the association of disease with overweight and obesity in Japan and the US indicated that the associations were stronger in the US than in Japanese women and that there was no association in Japanese men [40]. Links between social, demographic, and behavioral risk factors for obesity also differ markedly in Japan, Korea, and the US [41]. The availability of biomarker data from Taiwan—a middle-income country undergoing rapid economic growth, increasing obesity, and with life expectancy recently increasing to levels similar to that of the US and England—allows for investigation of the biological risk associated with obesity in a population characterized by very different cultural, behavioral, socioeconomic, and dietary parameters.

We examine how elevated weight and obesity (using an indicator that considers both BMI and waist circumference) relate to having levels defined as clinical risk for cardiovascular, metabolic, and inflammatory markers in three aging societies that are now relatively similar in life expectancy but that differ in the timing of the epidemiological transition and obesity epidemic, history of economic development, socioeconomic levels, general lifestyle habits, health behaviors, and health care systems: the US, England, and Taiwan. Finding differences in the relationship between obesity and indicators of physiological dysregulation in these three aging societies will clarify whether increases in weight gain are equally problematic across all countries.

## 2. Methods

### 2.1. Participants and Settings

We use data from three nationally representative samples: the US National Health and Nutrition Examination Survey (NHANES, 2003–2006; *N* = 3855), the English Longitudinal Study of Ageing (ELSA, 2004-2005; *N* = 9139), and the Social Environment and Biomarkers of Aging Study (SEBAS) in Taiwan (2000; *N* = 1023). These surveys collect information on demographics, as well as anthropometric, physical, and biological measures.

NHANES regularly monitors the health and nutritional status of the US population. Every year, approximately 5,000 individuals undergo detailed interviews and medical examinations, which include collection of several physiological measures. NHANES utilizes a complex sampling design, and when weights are applied, the sample is representative of the noninstitutionalized American population. We use the 2003–2006 data since NHANES data is released in two-year intervals, and this sample is centered on 2004-2005, which matches the period in which ELSA was collected. For NHANES, we use individual-level data based on a sample of 1,513 fasting individuals aged 54 and older.

ELSA includes participants drawn from households responding to the Health Survey for England (HSE) in 1998, 1999, and 2001 [42] and is representative of the English population aged 50 and older. The core ELSA sample (wave 1: 2002-2003) includes people living in an HSE responding household who were born prior to March 1, 1952, and their partners who could be under age 50. Wave 4 of ELSA (2008-2009), which includes a nurse visit, includes wave 1 core members, if they are still alive and do not refuse further contact after the first interview at wave 1. It also includes a refresher sample to maintain the age structure of the sample (in waves 3 and 4), and their partners. For ELSA, we use individual-level data based on a sample of 7,384 individuals aged 54 and older.

SEBAS is drawn from a follow-up survey of the Survey of Health and Living Status of the Near Elderly and Elderly in Taiwan (also known as the Taiwan Longitudinal Study of Aging (TLSA)), a nationally representative survey of Taiwanese adults (including institutionalized individuals) collected in 1989, 1993, 1996, 1999, and 2000. In 2000, a subsample of individuals was randomly selected for inclusion in SEBAS. SEBAS consists of adults aged 54 and older in 2000, with in-home interviews and medical exams taken in a hospital. For SEBAS, we use individual-level data based on a sample of 1,020.

The sample averages 66.8 years of age in England, and the US and Taiwan mean age is about the same (Table 1). There are more men in Taiwan (56%) and England (53%) and fewer in the US (44%). 

### 2.2. Measures

We examine the following indicators of physiological dysregulation often associated with obesity and also associated with increased risk for multiple adverse health outcomes and obesity [43, 44]: (1) cardiovascular markers: high systolic (SBP) and diastolic blood pressure (DBP); (2) metabolic markers: high levels of blood lipids (total and low-density lipoprotein (LDL) cholesterol, and fasting triglycerides), low levels of high-density lipoprotein (HDL) cholesterol, and high fasting glucose and glycated hemoglobin; (3) high levels of inflammatory markers C-reactive protein (CRP; available in NHANES and ELSA) and interleukin-6 (IL-6; available in SEBAS), as CRP and IL-6 have been positively associated with BMI [35]. For each indicator we use clinical cutpoints or widely used research-based cutpoints to indicate high levels of risk which are shown in Table 1 [39, 43, 45].

There has been debate as to the best indicator of obesity: body mass index (BMI) or waist circumference. Waist circumference is thought to be a better measure of abdominal adiposity than BMI and a better indicator of risk of poor health outcomes, including all-cause and cardiovascular mortality [46, 47]. For this reason we combine the two indicators in our investigation. We investigate the association between BMI and biomarkers across categories of BMI (underweight <18.5 kg/m^2^, normal and overweight 18.5–29.9 kg/m^2^, obese ≥30–34.9 kg/m^2^, and very obese ≥35 kg/m^2^) and waist circumference categorized as normal or high waist (high waist: men ≥120 cm, women ≥88 cm). We create a composite measure of obesity and adiposity by categorizing individuals into five groups: (1) underweight and normal waist (all underweight individuals had a normal waist circumference), (2) normal/overweight BMI (termed normal BMI) and normal waist circumference (reference group), (3) normal/overweight BMI (termed normal BMI) and high waist circumference (termed high waist), (4) obese and high waist (all obese individuals had a high waist circumference), and (5) very obese. We also evaluate an alternate definition for obesity in Taiwan based on BMI ≥27, as it has been suggested by some that obesity levels should be differentially defined for Asians [48, 49]. A similar composite measure of obesity and adiposity was calculated using this alternate definition of BMI in Taiwan.

Because these risk factors are all assumed to be associated with obesity and because dysregulation in multiple physiological systems has been shown to predict many of the poor health risk outcomes associated with aging, we also create two summary measures of risk based on the total number of at-risk levels of biomarkers, either 9 or 8 [50]. Because CRP values for SEBAS are not available, this measure is not included in the 9-item summary measure for Taiwan, but a summary measure (range 0–9) was calculated for Taiwan using IL-6, another indicator of inflammation, instead of the CRP values included for the US and England. A second alternate summary measure of biological risk, excluding the inflammatory marker (range 0–8), was examined for all three countries. Biological risk summary scores were computed for individuals who had missing values on no more than 3 biological markers.

We examine multiple covariates in our investigation of the relationship between obesity and biological risk. Self-reported use of antihypertensives was determined in all three countries, and use of lipid-lowering statins was only asked in the US sample. Dichotomous variables were created to indicate whether the respondent reported being a current smoker and participating in at least moderate physical activity for exercise (e.g., brisk walking, running, or swimming) in the past 30 days (for the US and England) or generally exercising once a week (for Taiwan). 

### 2.3. Statistical Analysis

We use logistic regression models to determine the odds of having at-risk levels of a specific biomarker for obese men and women among the three populations. For all countries, the comparison group for BMI and waist circumference is the normal BMI and normal waist group. The regressions included indicators of age, use of antihypertensives, current smoking status, and having exercised in the past 30 days. Ordinary least squares (OLS) regression models were used to determine the relationship between the summary measures of biological risk and the composite measure of obesity and adiposity. The OLS models were run with the same covariates as the logistic regression models.

## 3. Results

*Level of Physiological Dysregulation.* We begin by examining national differences in the high risk levels of individual biomarkers (Table 1). Elevated blood pressure is more prevalent in Taiwan than in England or the US. Low levels—or high risk levels—of HDL cholesterol are also more common in Taiwan. High total and LDL cholesterol is more common among the English; lower levels of plasma glucose, CRP, and glycated hemoglobin are also characteristic of the English. Few adults in England have elevated levels of fasting glucose (2.2%), while this is observed in 17.3% and 13.2% of American and Taiwanese adults.

*Levels of BMI and Waist Circumference.* In all countries, most people in this age range are in the normal to overweight category (64.7%, 67.2%, and 89.4% in the US, England, and Taiwan, respectively) (Table 2(a)). Americans are more likely to be obese (33.7%) compared to the English (32.1%) and Taiwanese (7.2%). Among the obese, Americans are much more likely to be very obese: 13.4% of the total US sample, 10.1% in England, and about 1% in Taiwan. Both among the obese and very obese, the average BMI is higher in the US and England compared to Taiwan. Few are underweight in any country (1.7%, 0.8%, and 3.4% in the US, England, and Taiwan, resp.). 

When we examine waist circumference, the US has the highest average waist circumference, with 65.5% of Americans, 55.9% of English, and 15.8% of Taiwanese having a high waist size (Table 2(a)). This means that high waist characterizes a substantial number of those who would be categorized as normal weight in the US and England. Among those in the normal and overweight group about half (49.5%) of Americans and a third (36.4%) of the English have high waist (Table 2(b)). Almost all obese individuals have a high waist in the US and England (98.3% in the US and 96.5% in England), but only 78.4% of the obese in Taiwan also have a high waist. When we use the alternate obese cutpoint of ≥27 kg/m^2^ in Taiwan, less than half of the obese individuals have a high waist (not shown here).

*Values of Control Variables.* Americans exhibit the highest proportion of the older population taking antihypertensive medication (47.1%) (Table 1). The percentage who reports taking antihypertensives is lower in England (32.0%) and Taiwan (28.6%). Americans are more likely to be current smokers (24.5%) than persons in Taiwan (22.5%) and England (13.9%). More than half of the population in all countries report having exercised in the past 30 days, with more English exercising (82.2%) compared to Taiwan (61.4%) and the US (58.5%).

*Links between Obesity and Physiological Dysregulation.* Men with normal BMI and high waist have a greater likelihood than men with normal BMI and normal waist size of having high-risk levels of triglycerides in all three countries. In the US and England, men with high waist are more likely to have high levels of glycosylated hemoglobin and higher CRP; fasting glucose is also elevated among this group in the US (Table 3(a)). Men who are obese in the US have fewer elevated risk factors than those with high waist who are not obese; in the US, obese men are only more likely than normal weight men without high waist to have elevated glycated hemoglobin, fasting glucose and CRP. Taiwanese obese men also have elevated glycated hemoglobin and high triglyceride levels. English men who are obese have more elevated risk: both blood pressure indicators, HDL cholesterol, triglycerides, glycated hemoglobin, and CRP. Very obese men in England have the same elevated risk factors with the exception of DBP. Very obese men in the US are more likely to have elevated fasting glucose in addition to CRP and glycated hemoglobin.

Results for women were somewhat different. English and Taiwanese women with normal weight and high waist are more likely to have elevated SBP, DBP, and glycosylated hemoglobin; only British women with higher waist have significantly elevated triglycerides and only the Taiwanese women had more HDL risk. High risk CRP is more common among both American and English women with normal BMI and high waist, and this risk of elevated CRP is also higher in the obese and very obese (Table 3(b)). Obese women in Britain had elevations in the same markers as normal BMI and high waist English women, while obese women in the US only have elevated fasting glucose, glycated hemoglobin, and CRP; obese women in Taiwan only had high DBP. With the exception of Taiwan, levels of the inflammatory markers (CRP in the US and England; IL-6 in Taiwan) are more likely to be elevated among persons with a high waist and normal BMI, obese or very obese, compared to their normal BMI and normal waist counterparts.

Among men, an increase in the biological risk summary score (range 0–9) is associated with having a high waist relative to being of normal BMI and normal waist in all three countries (Table 4(a)). Being obese or very obese is also related to a higher biological risk summary score (0–9) for men in the US and England. Generally, the size of the associated increase is larger with increasing weight. These equations explain 6 to 11 percent of the variance in the summary indicator of biological risk. These relationships are similar for women (Table 4(b)), with one exception: obese Taiwanese women do not have a significantly increased biological risk compared to their normal weight and normal waist counterparts.

When we consider the alternate summary score that excludes our indicators of inflammation (range 0–8), being obese or very obese is no longer associated with a higher biological risk summary score in US men compared to men with a normal BMI and normal waist when controls for health behaviors and medication use are included (Table 5(a)). The size of the effects of the obesity categories is reduced on the 8-indicator summary measure in both England and the US, indicating the strong link between CRP and obesity. The *R* ^2^ is also reduced in these equations for England and the US. For Taiwan, the 8 and 9 category measures yield very similar results.

Women in England and Taiwan, but not women in the US, with a higher waist but who are not obese have significantly higher physiological dysregulation compared to their normal BMI and normal waist counterparts. Obese women in all three countries have elevated risk and the very obese have even higher risk (Table 5(b)). The alternate biological risk summary measure (0–8) yields different relationships between weight and physiological dysregulation in the US and Taiwan. In the US, only very obese women have a higher alternate biological risk summary score (0–8). In Taiwan, obese and normal BMI and high waist women exhibit higher alternate biological risk summary scores compared to their normal BMI and normal waist counterparts, except when smoking status, physical activity, and use of hypertensives are included. 

**Figure 1:**
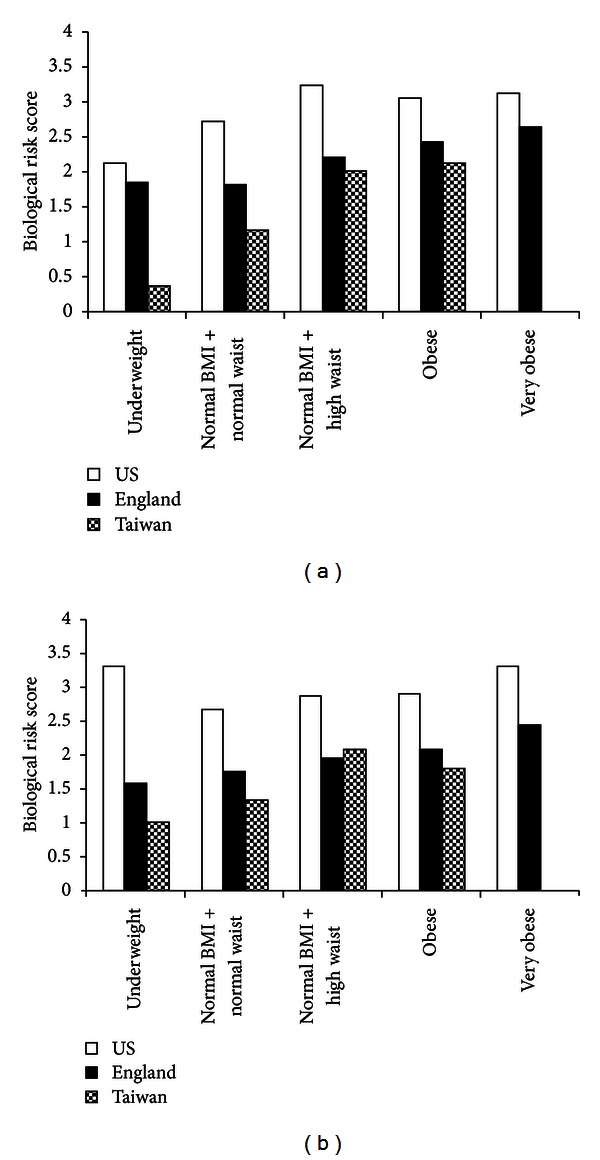
(a) Predicted biological risk summary measure (range: 0–8) in the US, England, and Taiwan by weight categories for men aged 65 who are nonsmokers, do not engage in physical activity, and are currently taking antihypertensive medication. (b) Predicted biological risk summary measure (range: 0–8) in the US, England, and Taiwan by weight categories for women aged 65 who are nonsmokers, do not engage in physical activity, and are currently taking antihypertensive medication.

Figures 1(a) and 1(b) illustrate the predicted alternate biological risk score (0–8) for each weight category for men and women (respectively) aged 65 who are nonsmokers, do not engage in physical activity, and are currently taking antihypertensive medication. Equations in Tables 5(a) and 5(b) are the basis of this estimation. This figure allows for country comparisons of individuals with these characteristics within each weight category and across weight categories. Generally, increases in weight categories correspond to an increase in biological risk score. The effect of weight appears to be largest among Taiwanese men. Across countries, the predicted values indicate that the US has the highest biological risk score within each respective weight category among men and women of the same age and lifestyle behaviors. With the exception of women in the normal BMI and high waist group, England has the second highest biological risk score within each weight category, followed by Taiwan. Among 65-year-old women with the noted lifestyle behaviors and with a normal BMI and high waist, Taiwan has a slightly higher biological risk score than England.

When we consider the alternate BMI cutpoint for obesity in Taiwan (BMI ≥ 27 kg/m^2^), our findings for the individual biomarkers and summary measures of biological risk are similar to using the BMI ≥ 30 kg/m^2^ cutoff for Taiwan except that the category normal weight with high waist no longer differs from the omitted category (results not shown).

## 4. Discussion

This study observes three general findings about how biological risk is associated with obesity in three countries that differ in lifestyle and culture. First, obesity is associated with physiological dysregulation in all countries with differences in the links between specific indicators of biological risk and obesity. Generally, obesity in England is associated with hypertension, dyslipidemia, and elevated glycated hemoglobin; Americans who are obese are not more likely to have hypertension. In Taiwan, obese women are more likely to have elevated DBP and obese men have an increased risk of elevated triglycerides and glycated hemoglobin compared to their nonobese, normal waist counterparts. Our biological risk summary scores indicate that at all levels of weight physiological dysregulation was highest in the US, followed by England (with one exception), with Taiwanese exhibiting the lowest biological risk in all groups among the three countries. Second, these relationships remain after controlling for demographic factors, participation in physical activity, and other behavioral factors. Third, similar to obese older adults, high waist individuals with normal BMI also exhibit greater physiological dysregulation in all countries compared to their normal BMI and normal waist counterparts. This dysregulation appears to be largest in Taiwan. There are, however, noted gender differences across the countries. Obesity in US men appeared to have a somewhat smaller effect on physiological dysregulation.

The country differences in the links between obesity and biological risk are particularly interesting. Our finding of a higher physiological dysregulation, as shown by the alternate biological risk summary score, in Taiwan compared to the US and England could be due to a couple of potential explanations. First, it may be due to differences in the years lived with obesity. The prevalence of obesity in the US and England is much higher than in Taiwan, indicating an earlier initial rise in obesity relative to Taiwan. From 1978 to 2002, the proportion of obese Americans and Britons exhibited stark increases (13–32% and 6–23% for men and women, resp.) [26]. The estimates for obesity prevalence in Taiwan indicate a recent increase for men but not women. From 1993–1996 to 2000-2001, the age-adjusted prevalence of obesity rose from 10.5% to 15.9% for men and declined from 13.2% to 10.7% in women [51]. It may be that the lower levels of risk among older adults who have lived longer years with obesity could be a reflection of better pharmacologic control of physiological dysregulation (e.g., through statin use), which may in turn confer less biological risk in these populations compared to populations of currently obese Taiwanese adults who may have more recently begun living with obesity.

A second reason for the observed country differences in obesity may be due to differences in dietary habits and lifestyle. The US and England are two modern, Western populations whose diets have been influenced by increased industrialization and have over time come to be characterized by high glycemic loads and high fatty acid composition [52]. Taiwan, on the other hand, represents a country that has experienced the effects of the industrial and scientific revolutions later than that of the US and England but is currently rapidly undergoing economic development and demographic change. The recent economic changes in Taiwan may indicate that obese older adults in Taiwan have more recently begun to consume high-fat diets, which could result in greater initial physiological dysregulation associated with access to Western-influenced dietary habits.

Despite controlling for lifestyle behaviors thought to be linked with health, the country differences in obesity and physiological regulation remain. Moreover, the consideration of antihypertensives does not alter our substantive conclusions on these associations. This suggests that despite the greater use of medications to treat hypertension in the US, obesity among Americans is associated with greater overall biological risk than the other two countries. This is supported by findings from the general population of Americans relative to England, which report that the US is faced with greater health disadvantages than England in adulthood [36] and across the life span [38].

We also note differences in biological profiles of obese individuals between the two Westernized countries: the US and England. The excess risk of hypertension associated with obesity in England was not found in the US. These differences may be due to the higher use of medications among Americans compared to the English, with about 16% more men and 18% more women in the US aged 65+ taking antihypertensive medication compared to their British counterparts [53]. The greater use of hypertensive medications in the US is also noted when compared to Japan and countries across Europe [53]. 

Two notable differences in country patterns of the relationship between obesity and physiological dysregulation by sex are found. Among men in England and Taiwan, the order of magnitude of physiological dysregulation increases with higher weight categories; however, this is not observed for US men. This difference may be due to our inability to consider statin use in England and Taiwan, which may be particularly important in the relationship between obesity and physiological dysregulation for men. Conversely, the importance of considering statin use may be less vital to understanding the country differences in the association between obesity and physiological dysregulation among women, given that the relationship for women is more consistent across countries, namely in the US and England.

Differences in underweight and physiological dysregulation are also observed. In US women, underweight corresponds with higher biological risk (though nonsignificant) compared to women with normal BMI and normal waist. Underweight among men in Taiwan is significantly associated with much lower biological risk than their normal BMI and normal weight counterparts. Further studies will be required to explore possible explanations for these differences in physiological dysregulation.

The higher biological risk observed among normal BMI and high waist individuals relative to normal BMI and normal waist older adults builds upon previous studies that report on alternate indicators of body shape, which vary across countries [54]. The importance of waist circumference is underscored by our current study, as well as a growing body of literature on the predictive value of waist circumference on indicators of health. Higher rates of diabetes among older Americans compared to Britons have been accounted for by high waist circumference as opposed to BMI differences [30]. Additionally, increasing waist circumference is more predictive of greater risk of incident diabetes than BMI in middle-aged British men ([55] and the European Prospective Investigation into Cancer and Nutrition (EPIC)-Potsdam study [56]). Waist circumference, as an indicator of central fat mass, is thought to be more strongly associated with disease risk, and in our case with physiological dysregulation, compared to BMI, which is considered a cruder index of adiposity. Banks and colleagues [30] cite differences in physical activity, diet and greater psychosocial environmental challenges in America compared to England as potential mechanisms linking central adiposity and type 2 diabetes. Our study considers some of these possible mechanisms (e.g., physical activity and antihypertensive use) but finds that they explain little of the relationship between biological risk and adiposity among the three countries. Together, these results highlight the importance of considering waist circumference in investigating the links between indicators of health and adiposity. They also support the need for identifying additional mechanisms that explain these relationships.

Our finding of the biological risks associated with obesity among older Taiwanese adults underscores the growing concern for risks associated with obesity in countries rapidly undergoing modernization. In comparing the biological risk of obese individuals among the three countries, we are able to use these international comparisons to our advantage to examine how differences in modernization influence the health of older adults in different populations. This may have potential health policy implications that underscore the importance of addressing and controlling the rising obesity epidemic that has become most widespread in countries, like the US and England, that have long experienced high economic growth and in countries currently undergoing rapid economic development. The increasing use of biological information to inform our understanding of health represents an innovative method in biodemography that will further contribute to the testing of current comparative theory and the potential creation of new paradigms surrounding the influence of modernization on health.

There are a number of principal strengths of the current study. First is the use of a broad range of biological markers across three large-scale population surveys. The inclusion of biological information as objective precursors of health allows, to some extent, a fairly comparable comparison of indicators of health across the different populations. An exception to this uniform comparison of biomarkers across the three surveys is our use of inflammatory marker CRP in the US and England and our inclusion of a different marker of inflammation (IL-6) in Taiwan. Of note, CRP seems to be more strongly associated with obesity than IL-6. Second, our study considers two indicators of obesity: BMI and waist circumference. A growing body of literature has made distinctions between BMI and waist circumference, namely, suggesting that waist circumference is a better indicator of abdominal obesity, which in turn has been associated with obesity-related health risks. Our findings generally report a similar association between increased biological risk and (1) normal BMI and high waist and (2) obese and high waist. Using the US NHANES, [57] reported that when both waist circumference and BMI were included in their analyses, only waist circumference was a significant predictor of comorbidity. Although this and other studies have suggested that waist circumference may be a better indicator of obesity and risk for adverse health outcomes, our study finds the two indicators to be similarly associated with biological risk across the three countries.

We note some limitations of the current study. First, we examine population-based data from three countries at a single time point. This limits our ability to distinguish among age-period-cohort effects. Future studies of longitudinal data will allow for further investigations of the potential role of obesity on biological risk observed in the current associations. Second, we do not have measures of some lifestyle and medical behaviors for some of the datasets (e.g., statin use), which likely influence the relationship between obesity and biological risk. As such, we are unable to include such factors in our analyses of all three countries. It is possible that these lifestyle behaviors are key explanatory factors to the noted cross-country differences in obesity-related biological risk.

The cross-country differences in the relationship between increased biological risk for individuals who are obese and have a high waist underscore potential differences in health and lifestyle behaviors. These behaviors may be a result of country differences in economic development that we are not able to observe in this study. The country differences in the links between obesity and physiological dysregulation are particularly marked when comparing obesity among Taiwanese older adults relative to Westernized populations, such as the US and England. Further examination of these relationships over time and across other countries will contribute to our understanding of the potential factors responsible for these country-specific variations in biological risk, as obesity becomes increasingly more prevalent and older adults in various countries live more years with obesity and increased adiposity.

## Figures and Tables

**Table 1:** Characteristics of persons aged 54+ in the US, England, and Taiwan.

	US	England	Taiwan
*N *	1513	7384	1020
Age yrs. (mean)	66.3	66.8	66.4
Men (%)	43.7	52.8	56.5
Currently smoking* (%)	24.5	13.9	22.5
Physical activity (%)	58.5	82.2	61.4
Taking antihypertensive meds (%)	47.1	32.0	28.6
At-risk levels of biomarkers (%)			
Systolic blood pressure (≥140 mm Hg)	29.1	32.9	42.9
Diastolic blood pressure (≥90 mm Hg)	5.2	8.2	24.2
Total cholesterol (≥240 mg/dL)	17.6	27.3	14.6
High-density lipoprotein cholesterol (<40 mg/dL)	16.2	9.4	27.4
Low-density lipoprotein cholesterol (≥160 mg/dL)	11.9	18.6	17.1
Triglycerides (≥200 mg/dL)	23.6	14.7	10.6
Fasting glucose (≥126 mg/dL)	17.3	2.2	13.2
C-Reactive protein (≥3 mg/L)	39.9	35.0	
Interleukin-6 (≥4.64 pg/mL)			6.4
Glycated hemoglobin (≥6.4%)	13.0	12.8	15.3
Summary risk (mean; range: 0–9)	3.0	2.4	1.7
Summary risk (mean; range: 0–8)	2.6	2.0	1.7

*In Taiwan, this indicates having smoked in the past 6 months.

**Table:** (a)

		BMI categories (kg/m^2^)											Waist circumference (cm)	
		Underweight			Normal			Obese			Very obese		High	
		<18.5			18.5–29.9			30–34.9			≥35		≥102 males; ≥88 females	
	*N *	%	Mean ± SD		%	Mean ± SD		%	Mean ± SD		%	Mean ± SD	%	Mean ± SD
US	1490	1.7	17.3 ± 1.3		64.7	25.6 ± 2.9		20.3	32.1 ± 2.3		13.4	39.0 ± 4.4	65.5	109.5 ± 12.5
England	7071	0.8	17.4 ± 0.9		67.2	25.6 ± 2.5		22.0	32.0 ± 1.4		10.1	39.2 ± 4.5	55.9	97.5 ± 13.4
Taiwan	1019	3.4	17.5 ± 0.7		89.4	24.2 ± 2.7		6.3	31.6 ± 1.3		0.9	37.2 ± 2.2	15.8	85.4 ± 9.5

**Table:** (b)

BMI categories (kg/m^2^)	US		England		Taiwan	
	*N *	% with high waist	*N *	% with high waist	*N *	% with high waist
Underweight (<18.5)	20	0.0	49	0.0	40	0.0
Normal (18.5–29.9)	950	49.5	4786	36.4	910	11.1
Obese (30–34.9)	293	98.3	1503	96.5	60	78.4
Very obese (≥35)	174	100.0	653	99.7	9	100.0

*High waist circumference: men ≥ 102 cm; women ≥ 88 cm.

**Table:** (a) Men

	*N*			Normal BMI + high waist		
	US	England	Taiwan	US	England	Taiwan
				OR (95% CI)	OR (95% CI)	OR (95% CI)
Cardiovascular risk factors						
SBP measured	1183	3069	587	1.54 (1.03–2.31)	1.24 (1.01–1.53)	3.36 (0.81–13.91)
DBP measured	1183	3069	587	0.97 (0.37–2.53)	1.42 (1.00–2.04)	0.95 (0.21–4.44)
Metabolic risk factors						
Total-C	1181	2481	586	0.81 (0.46–1.43)	1.09 (0.81–1.46)	0.76 (0.09–6.31)
HDL-C	1181	2479	586	1.98 (1.22–3.20)	1.86 (1.35–2.55)	1.97 (0.58–6.77)
LDL-C	567	2410	584	1.13 (0.52–2.45)	1.26 (0.91–1.73)	1.25 (0.26–6.06)
Triglycerides	587	1543	586	2.35 (1.21–4.57)	2.52 (1.68–3.76)	5.71 (1.54–21.13)
Fasting glucose	585	1537	586	3.08 (1.52–6.26)	2.22 (0.92–5.35)	2.23 (0.54–9.17)
Glycated hemoglobin	1183	2455	586	2.71 (1.56–4.71)	1.62 (1.13–2.32)	2.00 (0.43–9.26)
Inflammation markers						
C-Reactive protein	1181	2481		2.35 (1.58–3.49)	1.77 (1.39–2.25)	
Interleukin-6			586			∗

	Obese			Very obese		
	US	England	Taiwan	US	England	Taiwan
	OR (95% CI)	OR (95% CI)	OR (95% CI)	OR (95% CI)	OR (95% CI)	OR (95% CI)

Cardiovascular risk factors						
SBP measured	1.00 (0.64–1.56)	1.53 (1.24–1.89)	1.68 (0.75–3.78)	0.97 (0.54–1.75)	1.71 (1.25–2.34)	
DBP measured	1.19 (0.50–2.87)	1.57 (1.11–2.22)	1.30 (0.53–3.18)	0.46 (0.15–1.41)	1.50 (0.94–2.39)	
Metabolic risk factors						
Total-C	1.06 (0.58–1.93)	1.05 (0.79–1.41)	0.78 (0.21–2.90)	1.76 (0.86–3.61)	0.81 (0.50–1.31)	
HDL-C	1.60 (0.97–2.64)	2.4 (1.75–3.30)	2.19 (0.94–5.07)	1.71 (0.89–3.27)	3.18 (2.08–4.87)	
LDL-C	0.34 (0.12–0.96)	1.09 (0.77–1.54)	1.13 (0.34–3.73)	1.35 (0.40–4.60)	0.83 (0.49–1.43)	
Triglycerides	1.75 (0.85–3.60)	3.68 (2.50–5.41)	5.31 (1.91–14.73)	1.64 (0.65–4.16)	4.03 (2.33–6.95)	
Fasting glucose	2.23 (1.00–5.02)	2.00 (0.73–5.47)	2.53 (0.89–7.24)	3.57 (1.40–9.10)	2.73 (0.76–9.87)	
Glycated hemoglobin	2.37 (1.31–4.30)	2.38 (1.67–3.39)	4.48 (1.71–11.72)	4.25 (2.19–8.27)	5.07 (3.23–7.95)	
Inflammation markers						
C-Reactive protein	2.21 (1.44–3.39)	2.21 (1.72–2.85)		3.40 (1.96–5.89)	4.30 (2.94–6.29)	
Interleukin-6			1.04 (0.13–8.57)			

*Could not be estimated.

SBP: systolic blood pressure, DBP: diastolic blood pressure, Total-C: total cholesterol, HDL-C: high-density lipoprotein cholesterol, LDL-C: low-density lipoprotein cholesterol; OR: odds ratio; CI: confidence interval.

All models adjusted for age, use of antihypertensives, smoking status, and physical activity.

High-waist circumference men ≥ 102 cm, women ≥ 88 cm.

Referent group: normal body mass index (18.5–29.9 kg/m^2^) + normal waist.

The results are not computed for Very Obese in Taiwan because too few individuals are classified as very obese.

**Table:** (b) Women

	*N*			Normal BMI + high waist		
	US	England	Taiwan	US	England	Taiwan
				OR (95% CI)	OR (95% CI)	OR (95% CI)
Cardiovascular risk factors						
SBP measured	707	3721	431	0.90 (0.56–1.47)	1.46 (1.20–1.78)	2.00 (1.14–3.49)
DBP measured	707	3721	431	0.72 (0.26–2.04)	1.72 (1.17–2.53)	2.49 (1.34–4.63)
Metabolic risk factors						
Total-C	721	3029	431	1.60 (0.92–2.80)	0.96 (0.78–1.17)	1.15 (0.61–2.19)
HDL-C	721	3026	431	1.19 (0.42–3.37)	1.81 (0.96–3.40)	1.98 (1.09–3.60)
LDL-C	349	2986	431	2.25 (0.78–6.44)	1.07 (0.85–1.34)	0.99 (0.51–1.91)
Triglycerides	361	1879	431	1.16 (0.49–2.74)	3.15 (2.02–4.93)	1.79 (0.85–3.77)
Fasting glucose	366	1861	431	4.96 (1.64–14.98)	9.82 (1.23–78.48)	1.83 (0.94–3.57)
Glycated hemoglobin	731	2993	430	2.02 (0.85–4.81)	2.07 (1.34–3.22)	2.03 (1.11–3.71)
Inflammation markers						
C-Reactive protein	723	3029		2.02 (1.23–3.32)	2.03 (1.62–2.55)	
Interleukin-6			431			1.12 (0.38–3.30)

	Obese			Very obese		
	US	England	Taiwan	US	England	Taiwan
	OR (95% CI)	OR (95% CI)	OR (95% CI)	OR (95% CI)	OR (95% CI)	OR (95% CI)

Cardiovascular risk factors						
SBP measured	1.16 (0.66–2.04)	1.53 (1.24–1.90)	2.02 (0.90–4.53)	1.23 (0.64–2.40)	2.17 (1.67–2.82)	
DBP measured	1.16 (0.38–3.54)	2.06 (1.39–3.05)	2.41 (1.13–5.13)	1.19 (0.36–3.96)	3.44 (2.22–5.32)	
Metabolic risk factors						
Total-C	1.18 (0.60–2.33)	0.82 (0.65–1.02)	1.30 (0.59–2.88)	1.32 (0.65–2.68)	0.67 (0.49–0.91)	
HDL-C	2.88 (1.02–8.14)	1.71 (0.86–3.38)	0.99 (0.42–2.37)	2.89 (1.06–7.92)	4.66 (2.38–9.13)	
LDL-C	1.57 (0.46–5.40)	1.01 (0.78–1.32)	0.90 (0.38–2.09)	1.84 (0.53–6.33)	0.96 (0.68–1.34)	
Triglycerides	1.42 (0.57–3.56)	3.36 (2.10–5.37)	1.08 (0.40–2.92)	1.36 (0.51–3.64)	5.94 (3.54–9.97)	
Fasting glucose	3.32 (1.01–10.87)	10.06 (1.21–83.81)	1.33 (0.56–3.15)	13.28 (4.23–41.66)	37.51 (4.71–298.77)	
Glycated hemoglobin	3.47 (1.41–8.52)	3.65 (2.37–5.62)	1.52 (0.69–3.36)	9.05 (3.92–20.87)	7.00 (4.40–11.12)	
Inflammation markers						
C-Reactive protein	4.93 (2.79–8.72)	4.18 (3.29–5.32)		10.08 (5.11–19.89)	8.61 (6.24–11.88)	
Interleukin-6			1.05 (0.30–3.68)			

SBP: systolic blood pressure, DBP: diastolic blood pressure, Total-C: total cholesterol, HDL-C: high-density lipoprotein cholesterol, LDL-C: low-density lipoprotein cholesterol; OR: odds ratio; CI: confidence interval.

All models adjusted for age, use of antihypertensives, smoking status, and physical activity.

High-waist circumference men ≥ 102 cm, women ≥ 88 cm.

Referent group = normal body mass index (18.5–29.9 kg/m^2^) + normal waist.

The results are not computed for Very Obese in Taiwan because too few individuals are classified as very obese.

**Table:** (a) Men

	Summary measure (0–9)^||^							
	US (*N* = 995)		England (*N* = 2385)		Taiwan (*N* = 586)		Taiwan (*N* = 586)^∂^	
	Model 1	Model 2	Model 1	Model 2	Model 1	Model 2	Model 1	Model 2
Intercept	2.51*	3.18*	0.53*	0.60*	1.86*	2.67*	1.52*	2.31*
Age	0.00	−0.01	0.02*	0.02*	0.00	−0.01	0.00	−0.01
Currently smoking		0.25		0.42*		0.12		0.14
Physical activity		−0.51*		−0.25*		0.12		0.15
Antihypertensives		0.25		−0.013		−0.73*		−0.72*
BMI and waist circumference								
Underweight^*◊*^	−0.56	−0.39	0.44	0.21	−0.94*	−0.78*	−0.90*	−0.73*
Normal BMI + normal waist	Reference		Reference		Reference		Reference	
Normal BMI + high waist	0.73*	0.69*	0.51*	0.50*	0.85*	0.78*	0.29	0.27
Obese^†^	0.53*	0.51*	0.78*	0.78*	0.98*	0.96*	0.69*	0.67*
Very obese^†^	0.88*	0.67*	1.14*	1.14*				

*R* ^ 2^	0.04	0.06	0.07	0.09	0.05	0.10	0.06	0.11

**Table:** (b) Women

	Summary measure (0–9)^||^							
	US (*N* = 614)		England (*N* = 2918)		Taiwan (*N* = 431)		Taiwan (*N* = 431)^∂^	
	Model 1	Model 2	Model 1	Model 2	Model 1	Model 2	Model 1	Model 2
Intercept	2.46*	2.32*	−0.86*	−0.91*	0.22	1.61*	0.10	1.47*
Age	0.00	0.00	0.04*	0.04*	0.02*	0.01	0.02*	0.01
Currently smoking		0.23		0.36*		−0.02		0.04
Physical activity		−0.12		−0.03		0.04		0.08
Antihypertensives		0.36*		0.12		−0.92*		−0.91*
BMI and waist circumference								
Underweight^*◊*^	0.37	0.64	−0.06	−0.16	−0.49	−0.16	−0.45	−0.13
Normal BMI + normal waist	Reference		Reference		Reference		Reference	
Normal BMI + high waist	0.39	0.33	0.35*	0.34*	0.84*	0.75*	1.15*	1.04*
Obese^†^	0.59*	0.57*	0.67*	0.65*	0.69*	0.47	0.59*	0.44*
Very obese^†^	1.28*	1.13*	1.19*	1.16*				

*R* ^ 2^	0.05	0.06	0.13	0.14	0.08	0.15	0.09	0.16

BMI categories: underweight < 18.5 kg/m^2^, normal 18.5–29.9 kg/m^2^, obese ≥ 30 kg/m^2^, very obese ≥ 35 kg/m^2^ (the results are not computed for Taiwan because too few individuals are classified as very obese).

^∂^These models for Taiwan group BMI categories: underweight < 18.5 kg/m^2^; normal 18.5–26.9 kg/m^2^; obese ≥ 27 kg/m^2^.

Model 1 includes age and BMI and waist circumference category.

Model 2 includes Model 1 covariates in addition to smoking, physical activity, and antihypertensives.

^◊^All underweight individuals had a normal waist circumference.

^†^All obese individuals had a high waist circumference.

^||^Includes CRP (for US and England) and IL-6 (for Taiwan); *P < .05.

**Table:** (a) Men

	Summary measure (0–8)^‡^							
	US (*N* = 995)		England (*N* = 2385)		Taiwan (*N* = 586)		Taiwan (*N* = 586)^∂^	
	Model 1	Model 2	Model 1	Model 2	Model 1	Model 2	Model 1	Model 2
Intercept	2.36*	3.31*	0.53*	0.61*	1.76*	2.51*	1.43*	2.16*
Age	0.00	−0.01	0.02*	0.02*	0.00	−0.01	0.00	−0.01
Currently smoking		0.01		0.24*		0.12		0.14
Physical activity		−0.45*		−0.18		0.15		0.17
Antihypertensives		0.20		−0.02		−0.68*		−0.67*
BMI and waist circumference								
Underweight^*◊*^	−0.81	−0.59	0.18	0.03	−0.96*	−0.80*	−0.91*	−0.76*
Normal BMI + normal waist	Reference		Reference		Reference		Reference	
Normal BMI + high waist	0.56*	0.52*	0.38*	0.38*	0.90*	0.84*	0.35	0.33
Obese^†^	0.34	0.34	0.60*	0.61*	0.98*	0.96*	0.67*	0.65*
Very obese^†^	0.62*	0.40	0.82*	0.82*				

*R* ^ 2^	0.03	0.04	0.05	0.06	0.05	0.10	0.06	0.11

**Table:** (b) Women

	Summary measure (0–8)^‡^							
	US (*N* = 614)		England (*N* = 2918)		Taiwan (*N* = 431)		Taiwan (*N* = 586)^∂^	
	Model 1	Model 2	Model 1	Model 2	Model 1	Model 2	Model 1	Model 2
Intercept	2.31*	2.27*	−0.93*	−1.10*	0.30	1.66*	0.18	1.53*
Age	0.00	0.00	0.04*	0.04*	0.02*	0.01	0.02*	0.01
Currently smoking		0.14		0.27*		−0.14		−0.08
Physical activity		−0.06		0.05		0.07		0.10
Antihypertensives		0.39*		0.10		−0.92*		−0.91*
BMI and waist circumference								
Underweight^*◊*^	0.44	0.63	−0.08	−0.16	−0.66	−0.32	−0.62	−0.30
Normal BMI + normal waist	Reference		Reference		Reference		Reference	
Normal BMI + high waist	0.26	0.19	0.22*	0.21*	0.84*	0.74*	1.12*	1.01*
Obese^†^	0.26	0.22	0.35*	0.34*	0.69*	0.47	0.58*	0.43*
Very obese^†^	0.80*	0.63*	0.70*	0.69*				

*R* ^ 2^	0.02	0.03	0.10	0.11	0.08	0.15	0.09	0.16

BMI categories: underweight < 18.5 kg/m^2^; normal 18.5–29.9 kg/m^2^; obese ≥ 30 kg/m^2^; very obese ≥ 35 kg/m^2^ (the results are not computed for Taiwan because too few individuals are classified as very obese).

^∂^These models for Taiwan group BMI categories: underweight < 18.5 kg/m^2^; normal 18.5–26.9 kg/m^2^; obese ≥ 27 kg/m^2^.

Model 1 includes age and BMI and waist circumference category.

Model 2 includes Model 1 covariates in addition to smoking, physical activity, and antihypertensives.

^◊^All underweight individuals had a normal waist circumference.

^†^All obese individuals had a high waist circumference.

^‡^Excludes CRP (for US and England) and IL-6 (for Taiwan); *P < .05.

## References

[1] Deitel M (2003). Overweight and obesity worldwide now estimated to involve 1.7 Billion people. *Obesity Surgery*.

[2] James PT (2004). Obesity: the worldwide epidemic. *Clinics in Dermatology*.

[3] James PT, Leach R, Kalamara E, Shayeghi M (2001). The worldwide obesity epidemic. *Obesity Research*.

[4] Popkin BM, Doak CM (1998). The obesity epidemic is a worldwide phenomenon. *Nutrition Reviews*.

[5] Eliasson M, Lindahl B, Lundberg V, Stegmayr B (2003). Diabetes and obesity in Northern Sweden: occurrence and risk factors for stroke and myocardial infarction.. *Scandinavian journal of public health*.

[6] Hubert HB, Feinleib M, McNamara PM, Castelli WP (1983). Obesity as an independent risk factor for cardiovascular disease: a 26-year follow-up of participants in the Framingham Heart Study. *Circulation*.

[7] McKeigue PM, Shah B, Marmot MG (1991). Relation of central obesity and insulin resistance with high diabetes prevalence and cardiovascular risk in South Asians. *The Lancet*.

[8] Moon OR, Kim NS, Jang SM, Yoon TH, Kim SO (2002). The relationship between body mass index and the prevalence of obesity-related diseases based on the 1995 National Health Interview Survey in Korea. *Obesity Reviews*.

[9] Must A, Spadano J, Coakley EH, Field AE, Colditz G, Dietz WH (1999). The disease burden associated with overweight and obesity. *Journal of the American Medical Association*.

[10] Patterson RE, Frank LL, Kristal AR, White E (2004). A comprehensive examination of health conditions associated with obesity in older adults. *American Journal of Preventive Medicine*.

[11] Raymond SU, Leeder S, Greenberg HM (2006). Obesity and cardiovascular disease in developing countries: a growing problem and an economic threat. *Current Opinion in Clinical Nutrition and Metabolic Care*.

[12] Yusuf S, Hawken S, Ôunpuu S (2005). Obesity and the risk of myocardial infarction in 27 000 participants from 52 countries: a case-control study. *The Lancet*.

[13] Winter Y, Rohrmann S, Linseisen J (2008). Contribution of obesity and abdominal fat mass to risk of stroke and transient ischemic attacks. *Stroke*.

[14] Al Snih S, Ottenbacher KJ, Markides KS, Kuo YF, Eschbach K, Goodwin JS (2007). The effect of obesity on disability vs mortality in older Americans. *Archives of Internal Medicine*.

[15] Alley DE, Chang VW (2007). The changing relationship of obesity and disability, 1988–2004. *Journal of the American Medical Association*.

[16] Jenkins KR (2004). Obesity's effects on the onset of functional impairment among older adults. *Gerontologist*.

[17] Mokdad AH, Ford ES, Bowman BA (2003). Prevalence of obesity, diabetes, and obesity-related health risk factors, 2001. *Journal of the American Medical Association*.

[18] Stenholm S, Sainio P, Rantanen T (2007). High body mass index and physical impairments as predictors of walking limitation 22 years later in adult Finns. *Journals of Gerontology A Biological Sciences Medical Sciences*.

[19] James PT, Rigby N, Leach R (2004). The obesity epidemic, metabolic syndrome and future prevention strategies. *European Journal of Cardiovascular Prevention and Rehabilitation*.

[20] Katzmarzyk PT, Church TS, Janssen I, Ross R, Blair SN (2005). Metabolic syndrome, obesity, and mortality: impact of cardiorespiratory fitness. *Diabetes Care*.

[21] Sbarbati A, Osculati F, Silvagni D (2006). Obesity and inflammation: evidence for an elementary lesion. *Pediatrics*.

[22] Stienstra R, Duval C, Müller M, Kersten S (2007). PPARs, obesity, and inflammation. *PPAR Research*.

[23] Baik I, Ascherio A, Rimm EB (2000). Adiposity and mortality in men. *American Journal of Epidemiology*.

[24] Locher JL, Roth DL, Ritchie CS (2007). Body mass index, weight loss, and mortality in community-dwelling older adults. *Journals of Gerontology A*.

[25] Adams KF, Schatzkin A, Harris TB (2006). Overweight, obesity, and mortality in a large prospective cohort of persons 50 to 71 years old. *The New England Journal of Medicine*.

[26] Alley DE, Lloyd J, Shardell M, Crimmins EM, Preston SH, Cohen B (2011). Can obesity account for cross-national differences in life expectancy trends?. *International Differences in Mortality at Older Ages: Dimensions and Sources*.

[27] Prentice AM (2006). The emerging epidemic of obesity in developing countries. *International Journal of Epidemiology*.

[28] Fogel RW (2005). Changes in the physiology of aging during the twentieth century. *NBER Working Paper*.

[29] Fogel RW, Costa DL (1997). A theory of technophysio evolution, with some implications for forecasting population, health care costs, and pension costs. *Demography*.

[30] Banks J, Kumari M, Smith JP, Zaninotto P (2012). What explains the American disadvantage in health compared with the English? the case of diabetes. *Journal of Epidemiology and Community Health*.

[31] Lisko I, Tianien K, Stenholm S, Luukkaala T, Hervonen A, Jylha M (2011). Body mass index, waist circumference, and waist-to-hip ratio as predictors of mortality in nonagenarians: the Vitality 90+ Study. *Journals of Gerontology A Biological Sciences Medical Sciences*.

[32] Visscher TLS, Seidell JC, Molarius A, van der Kuip D, Hofman A, Witteman JCM (2001). A comparison of body mass index, waist-hip ratio and waist circumference as predictors of all-cause mortality among the elderly: the Rotterdam study. *International Journal of Obesity*.

[33] Andreyeva T, Michaud P-C, van Soest A (2007). Obesity and health in Europeans aged 50 years and older. *Public Health*.

[34] Grundy SM (2004). Obesity, metabolic syndrome, and cardiovascular disease. *Journal of Clinical Endocrinology and Metabolism*.

[35] Cesari M, Kritchevsky SB, Baumgartner RN (2005). Sarcopenia, obesity, and inflammation—results from the Trial of Angiotensin Converting Enzyme Inhibition and Novel Cardiovascular Risk Factors study. *American Journal of Clinical Nutrition*.

[36] Banks J, Marmot M, Oldfield Z, Smith JP (2006). Disease and disadvantage in the United States and in England. *Journal of the American Medical Association*.

[37] Banks J, Muriel A, Smith JP (2010). Disease prevalence, disease incidence, and mortality in the United States and in England.. *Demography*.

[38] Martinson ML, Teitler JO, Reichman NE (2011). Health across the life span in the United States and England. *American Journal of Epidemiology*.

[39] Crimmins E, Kim JK, Vasunilashorn S (2010). Biodemography: new approaches to understanding trends and differences in population health and mortality. *Demography*.

[40] Reynolds SL, Hagedorn A, Yeom J, Saito Y, Yokoyama E, Crimmins EM (2008). A tale of two countries-the United States and Japan: are differences in health due to differences in overweight?. *Journal of Epidemiology*.

[41] Yeom J, Kim JK, Crimmins E (2009). Factors associated with body mass index (BMI) among older adults: a comparison study of the US, Japan, and Korea. *Journal of the Korean Gerontological Society*.

[42] Marmot M, Banks J, Blundell R, Lessof C, Nazroo J (2003). *Health, Wealth and Lifestyles of the Older Population in England: ELSA 2002*.

[43] Crimmins E, Vasunilashorn S, Kim JK, Alley D (2008). Chapter 5 biomarkers related to aging in human populations. *Advances in Clinical Chemistry*.

[44] Garrison RJ, Wilson PW, Castelli WP (1980). Obesity and lipoprotein cholesterol in the Framingham Offspring Study. *Metabolism*.

[45] Crimmins EM, Vasunilashorn S, Kim JK, Hagedorn A, Saito Y (2008). A comparison of biological risk factors in two populations: the United States and Japan. *Population and Development Review*.

[46] Folsom AR, Kaye SA, Sellers TA (1993). Body fat distribution and 5-year risk of death in older women. *Journal of the American Medical Association*.

[47] Rimm EB, Stampfer MJ, Giovannucci E (1995). Body size and fat distribution as predictors of coronary heart disease among middle-aged and older US men. *American Journal of Epidemiology*.

[48] Goldman N, Glei DA, Lin Y-H, Weinstein M (2009). Improving mortality prediction using biosocial surveys. *American Journal of Epidemiology*.

[49] International Obesity Task Force (2000). *The Asia-Pacific Perspective: Redefining Obesity and Its Treatment*.

[50] Seeman TE, Crimmins E, Huang MH (2004). Cumulative biological risk and socio-economic differences in mortality: MacArthur Studies of Successful Aging. *Social Science and Medicine*.

[51] Chu NF (2005). Prevalence of obesity in Taiwan. *Obesity Reviews*.

[52] Cordain L, Eaton SB, Sebastian A (2005). Origins and evolution of the Western diet: health implications for the 21st century. *American Journal of Clinical Nutrition*.

[53] Crimmins EM, Garcia K, Kim JK, Crimmins EM, Preston SH, Cohen B (2011). Are international differences in health similar to international differences in life expectancy. *International Differences in Mortality at Older Ages*.

[54] Wells JCK, Cole TJ, Bruner D, Treleaven P (2008). Body shape in American and British adults: between-country and inter-ethnic comparisons. *International Journal of Obesity*.

[55] Perry IJ, Wannamethee SG, Walker MK, Thomson AG, Whincup PH, Shaper AG (1995). Prospective study of risk factors for development of non-insulin dependent diabetes in middle aged British men. *British Medical Journal*.

[56] Schulze MB, Heidemann C, Schienkiewitz A, Bergmann MM, Hoffmann K, Boeing H (2006). Comparison of anthropometric characteristics in predicting the incidence of type 2 diabetes in the EPIC-Potsdam study. *Diabetes Care*.

[57] Janssen I, Katzmarzyk PT, Ross R (2004). Waist circumference and not body mass index explains obesity-related health risk. *American Journal of Clinical Nutrition*.

